# Home Automated Telemanagement System for Individualized Exercise Programs: Design and Usability Evaluation

**DOI:** 10.2196/65734

**Published:** 2024-12-27

**Authors:** Aref Smiley, Joseph Finkelstein

**Affiliations:** 1 Department of Biomedical Informatics School of Medicine University of Utah Salt Lake City, UT United States

**Keywords:** telemedicine, home-based exercise, telerehabilitation, remote cycling, usability, physical rehabilitation, exercise therapy

## Abstract

**Background:**

Exercise is essential for physical rehabilitation, helping to improve functional performance and manage chronic conditions. Telerehabilitation offers an innovative way to deliver personalized exercise programs remotely, enhancing patient adherence and clinical outcomes. The Home Automated Telemanagement (HAT) System, integrated with the interactive bike (iBikE) system, was designed to support home-based rehabilitation by providing patients with individualized exercise programs that can be monitored remotely by a clinical rehabilitation team.

**Objective:**

This study aims to evaluate the design, usability, and efficacy of the iBikE system within the HAT platform. We assessed the system’s ability to enhance patient adherence to prescribed exercise regimens while minimizing patient and clinician burden in carrying out the rehabilitation program.

**Methods:**

We conducted a quasi-experimental study with 5 participants using a pre- and posttest design. Usability testing included 2 primary tasks that participants performed with the iBikE system. Task completion times, adherence to exercise protocols, and user satisfaction were measured. A System Usability Scale (SUS) was also used to evaluate participants’ overall experience. After an initial introduction, users performed the tasks independently following a 1-week break to assess retention of the system’s operation skills and its functionality.

**Results:**

Task completion times improved substantially from the pretest to the posttest: execution time for task 1 reduced from a mean of 8.6 (SD 4.7) seconds to a mean of 1.8 (SD 0.8) seconds, and the time for task 2 decreased from a mean of 315 (SD 6.9) seconds to a mean of 303.4 (SD 1.1) seconds. Adherence to the prescribed cycling speed also improved, with deviations from the prescribed speed reduced from a mean of 6.26 (SD 1.00) rpm (revolutions per minute) to a mean of 4.02 (SD 0.82) rpm (t=3.305, n=5, *P*=.03). SUS scores increased from a mean of 92 (SD 8.6) to a mean of 97 (SD 3.3), indicating high user satisfaction and confidence in system usability. All participants successfully completed both tasks without any additional assistance during the posttest phase, demonstrating the system’s ease of use and effectiveness in supporting independent exercise.

**Conclusions:**

The iBikE system, integrated into the HAT platform, effectively supports home-based telerehabilitation by enabling patients to follow personalized exercise prescriptions with minimal need for further training or supervision. The significant improvements in task performance and exercise adherence suggest that the system is well-suited for use in home-based rehabilitation programs, promoting sustained patient engagement and adherence to exercise regimens. Further studies with larger sample sizes are recommended to validate these findings and explore the long-term benefits of the system in broader patient populations.

## Introduction

Exercise is considered an essential component of physical rehabilitation [1]. Comprehensive disease management programs include regular exercise to improve cardiorespiratory fitness and quality of life. Physical rehabilitation involves the assessment, diagnosis, and treatment of patients with chronic health conditions and older adults with disabilities to restore their functional abilities and improve residual functions [2-4].

The telerehabilitation approach shows great potential to support home-based exercises. Successful implementation of home-based exercise interventions in patients using telerehabilitation technology should be tailored to individual patient’s needs, values, and preferences. Before considering telerehabilitation approaches for routine care, providers expect evidence of patient acceptance, adherence, and clinical impact of home-based exercise programs supported via telerehabilitation. The telerehabilitation approach can potentially facilitate the safe and effective use of exercise equipment in patient homes for rehabilitation. Despite heightened demand for telerehabilitation services [5], limited studies assessed the fidelity and usability of remotely managed exercise equipment for home-based telerehabilitation. Existing gaps in our knowledge of these approaches hinder their widespread introduction into routine clinical practices.

Remote patient monitoring applications have successfully supported self-care for various chronic health conditions [6-8]. Our studies have demonstrated the positive effects of interactive participation and engagement tools and the high acceptance of home telecare by patients [8,9]. We have also demonstrated the feasibility of home-based exercise telerehabilitation in patients with limited mobility [10].

Cycling exercise equipment is often used to facilitate upper and lower extremities training and is widely available in rehabilitation facilities that can oversee patient exercise [11]. It has been demonstrated that cycling exercise training improves clinical outcomes in individuals with chronic health disorders [12], chronic pulmonary disease [11], stroke recovery [13], mechanical ventilation [14], hip fracture [15], and those undergoing hemodialysis [16]. Cycling exercise equipment can be easily installed at patient homes. However, most exercise devices for home use lack remote connectivity with rehabilitation professionals, cannot communicate exercise progress in real time using simple graphics and numerical expressions, and do not provide warnings of insufficient or excessive levels of exertion to patients and remote rehabilitation teams.

A comprehensive telerehabilitation system supports patient-specific exercise activities tailored to individual’s health and fitness profiles, as assessed by rehabilitation professionals. Based on the patient’s specific needs and interests, the objectives of exercise prescription must be achieved by integrating exercise principles and behavioral techniques that motivate participants [11,13]. Telerehabilitation systems could help patients to follow their personalized rehabilitation programs. Previous reviews have demonstrated a significant increase in consumer health care applications to improve health and self-monitoring [17,18]. These applications were particularly efficient when adopting a patient-centric approach that describes the needs and personal goals of the patient [8,19]. Previous studies demonstrated high acceptance of home-based telemanagement applications by patients with varying chronic health conditions [7,20,21]. Individualized feedback and enhanced patient-provider communication supported regular exercise in home-based telerehabilitation programs [6,10]. To advance this approach, we developed a cycling exercise system that could be managed remotely and required minimal prior user background. It included functionality to support exercise in individuals with significant upper or lower limb disabilities. This interactive bike (iBikE) system consisted of a sensor for monitoring cycling exercise, algorithms for detecting exercise completion, programs for integrating the sensor with a mobile device such as a tablet, and user interfaces for displaying iBikE exercise progress and follow-up records. This iBikE system is novel because it can detect, monitor, and communicate user exercise records without structural constraints and support adaptable individualized exercise prescriptions that can be dynamically updated by remote rehabilitation teams. This study aims to assess adherence to the individually prescribed cycling speed using an iBikE that is remotely communicated by rehabilitation professionals. It seeks to introduce and evaluate a telerehabilitation system that requires minimal patient burden in performing daily exercise programs in a safe and effective manner by supporting home-based cycling exercises to provide maximum convenience to the patients.

We integrated the iBikE system as part of the Home Automated Telemanagement (HAT) system, a comprehensive closed-loop system to provide patient and clinician feedback based on patient exercise performance. In the HAT system, physicians can provide personalized exercise prescriptions for patients and follow their progress by reviewing the data stored in a database. The iBikE capabilities added to the HAT system support biking exercises at home. Patients can follow their exercise prescriptions and communicate with physicians. In this article, we aim to present the system design and structure of the HAT system and demonstrate the capability of the HAT system to support individualized exercise programs. In addition, we discuss the results of the iBikE system usability evaluation.

## Methods

### Overview

We designed and developed an interactive telerehabilitation system that supports a rehabilitation team’s prescription of individualized exercise plans. It also assists patients at home in following their individualized exercise program safely and effectively [22]. The iBikE functionality is an expansion of the HAT system, which was previously reported and has been found to effectively facilitate disease management in various chronic diseases [23-25]. We added our previously developed iBikE [26] to the HAT system in this new system. This study assessed the system’s capability to support biking at home and its usability.

### HAT System Design

The HAT system supports patient-centered care, such as guided patient self-management, individualized treatment plans, tailored education and counseling, decision support that complies with guidelines, thorough patient-provider communication, and multidisciplinary care coordination [27,28].

The HAT system includes a patient unit, a clinician unit, and an iBikE unit, which monitors rpm (revolutions per minute) through a magnetic sensor. Patient and clinician units were developed in .NET (.NET Foundation and the open-source community) using C# (Microsoft Corporation), and all data are stored in a SQL Server (Microsoft Corporation) database. Patient alerts are generated every night based on individually set up thresholds for exercise adherence, symptoms, or patient requests. The web-based interface supports establishing individualized alert thresholds for patient exercise completion, messages, daily log-ins, and postexercise question responses. A cadence sensor embedded into the portable bike unit for real-time speed monitoring is also connected to the HAT system. The sensor sends cadence data to a Windows-based application created in C#. The program connects with the SQL Server HAT database, obtains the user’s exercise prescription, and transmits the result.

**Figure 1:**
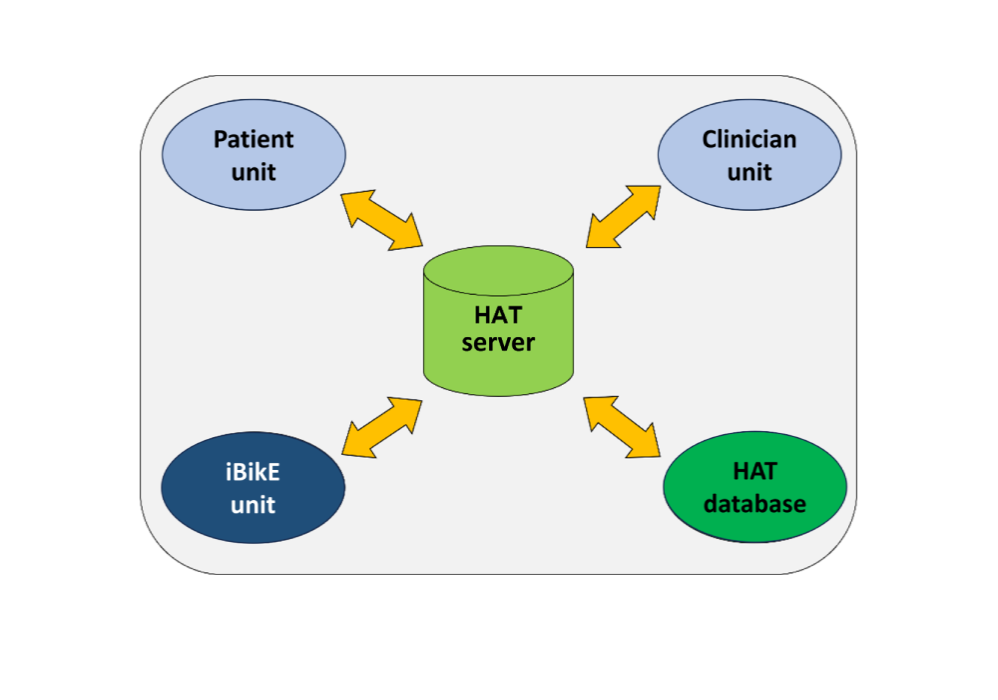
Home Automated Telemanagement (HAT) system architecture. This diagram presents the overall system architecture of the HAT system, including the interactions between the patient unit, clinician unit, and interactive bike (iBikE) unit for remote, personalized exercise monitoring and feedback. The patient unit allows individuals to access their prescribed exercise regimen via personal devices, while the clinician unit enables healthcare providers to monitor patient progress and adjust exercise prescriptions. The system supports geriatric rehabilitation for patients with chronic conditions, enabling home-based exercise programs. Data flow between units and the SQL server is illustrated.

The system architecture is displayed in Figure 1. The system architecture comprises a patient unit, a central data repository server, and a computerized disease management and care coordination unit. The patient unit web-based interface was designed with minimal requirements for its users. The server offers resources for configuring customized disease management plans, personalized patient notifications, and clinician care coordination. The system provides detailed exercise feedback to the patient rehabilitation team, allowing them to evaluate which exercise settings are optimal for the patient and adjust them in a timely manner. There are options for both general exercise safety advice and tips specific to each exercise to reduce the risk of injury while exercising (Figure 1).

Patients can access the system via a personal computer, a tablet, or a smartphone. A user-friendly interface enables patients with limited mobility, vision, or cognition to access the patient portal using a touch screen, mouse, keyboard, or speech, as the user desires. Providers could use the care management portal to set up and modify each patient’s exercise plan, monitor their progress, and communicate with them. The portal features a virtual prescription pad that enables physical therapists and exercise physiologists to prescribe activities individually, depending on the patient’s cardiorespiratory fitness evaluation. The individualized exercise program can be changed during the patient’s follow-up.

### Experimental Design

A quasi-experimental, single-group pre- and posttest design was used to evaluate the usability of the iBikE system as part of the HAT platform. Five participants were recruited for this study. The usability evaluation included 2 tasks (Textbox 1).

Each task was performed 2 times: first with guidance during the pretest and independently during the posttest following a 1-week break. Time to complete each task, adherence to the exercise performance protocol, and feedback were recorded during both sessions. A System Usability Scale (SUS) was administered after each task to assess user satisfaction (Table 1).

**User tasks for testing the interactive bike (iBikE) system (ie, the specific tasks participants were required to perform during the usability evaluation of the iBikE system).**

**1. Tasks performed by the users during the task analysis phase**

- Press the red button on the iBikE exercise equipment.
- Start the iBikE program for arm exercise and follow the prescribed cycling speed for 5 minutes.

**Table 1:** Posttask survey for the iBikEa System Usability Scale. This table presents the 3 questions the participants completed after each task. The participants were asked to rate their experience on a scale from 1 to 5, with 5 representing the most favorable response.

Questions asked after each task	Scale range	Subsession
1. How difficult or easy was it to complete this task?	1=very difficult to 5=very easy	Task X.1
2. How satisfied are you with using this application/system to complete this task?	1=very unsatisfied to 5=very satisfied	Task X.2
3. How would you rate the amount of time it took to complete this task?	1=too much time to 5=very little time	Task X.3

^a^iBikE: interactive bike.

### Data Collection and Analyses

Sociodemographic information and feedback on task difficulty, satisfaction, and time efficiency were collected using posttask surveys. Data were analyzed using SPSS 26 (IBM Corp.), with descriptive statistics such as means, SDs, and paired *t* tests (2-tailed) applied to compare pre- and posttest results.

### Usability Evaluation Protocol

Two usability evaluations were conducted at a 1-week interval for the usability assessment of the iBikE system. Users’ demographic data are provided in Table 2. The HAT assessment study consisted of 2 visits. During each visit, the steps mentioned in Textbox 2 were accomplished.

The task analysis session included the steps necessary to complete the 5-minute upper-body workout. Using SPSS 26 for Windows, a descriptive statistical analysis was performed, including mean values, SD, and frequency of categorical variables.

**Table 2:** Sociodemographic profile of participants in the iBikE^a^ system usability study. This table summarizes the demographic characteristics of the 5 participants who tested the iBikE system as part of the HAT^b^ system usability evaluation. Participants included adults with an average age of 37.4 (SD 14.2) years (all reported permanent jobs). The table also presents details on participants’ internet use, technology habits, and self-reported English proficiency.

User sociodemographic profile			Usability test (N=5)
**Age (years), mean (SD)**			37.4 (14.2)
**Gender, n (%)**			
	Male	4 (80)	
	Female	1 (20)	
**Race/ethnicity, n (%)**			
	Asian	4 (80)	
	Non-Hispanic white	1 (20)	
**Born in the continental United States, n (%)**			
	No	4 (80)	
	Yes	1 (20)	
**Job, n (%)**			
	Permanent	5 (100)	
**Internet use, n (%)**			
	Once a day	5 (100)	
**ATM use, n (%)**			
	Once a month or less	4 (80)	
	Never	1 (20)	
**Computer use at home, n (%)**			
	Once a day	5 (100)	
	Computer use at work/school, n (%)		
	Once a day	5 (100)	
**English proficiency (self-reported), n (%)**			
	Excellent	3 (60)	
	Good	2 (40)	
**How would you rate your proficiency in using the internet?, n (%)**			
	Excellent	5 (100)	

^a^iBikE: interactive bike.

^b^HAT: Home Automated Telemanagement.

**Steps accomplished during each visit.**

- Reference information (sociodemographics, computer familiarity, and health records) was collected.
- The interactive bike (iBikE) system was presented by a research assistant, who first introduced the tasks that users were required to perform (induction phase) and then asked the user to proceed with all the steps independently under the nonsupervising (task phase) situation. The time taken during the introductory phase was recorded using individual measurement software without the user’s awareness.
- In the task phase, the user was asked to use the system according to the tasks explained in the previous step without supervision to complete a telerehabilitation session consisting of a predefined series of tasks (the task analysis phase). While using the system, patients were instructed to speak up about the tasks. For each session, the time to complete each task, user opinions, and the ability to complete the task independently without prompting from the research assistant were documented. At the end of each task, patients grade it from 1 to 5 using a 3-item survey that includes the following questions: (1) How difficult or easy is it to complete this task? (2) How satisfied are you with using this application/system to complete this task? (3) How would you rate the time it took to complete this task? (4) After completing all tasks, an attitudinal survey measuring the patient’s attitude and system acceptance and a System Usability Scale was completed; and (5) the attitudinal and exit surveys were administered for additional input.

### Ethical Considerations

The study protocol was approved by the University of Utah IRB (IRB_00183926). All study participants signed an informed consent. Remuneration for completing surveys and providing feedback on the HAT system functionality was US $30. The resulting analytical data set was fully deidentified for further analyses.

## Results

### System Implementation

We successfully designed and implemented the HAT system for personalized biking exercises. The system consists of a patient unit, a clinician unit, an HAT server, and an iBikE unit.

### Patient Unit

**Figure 2:**
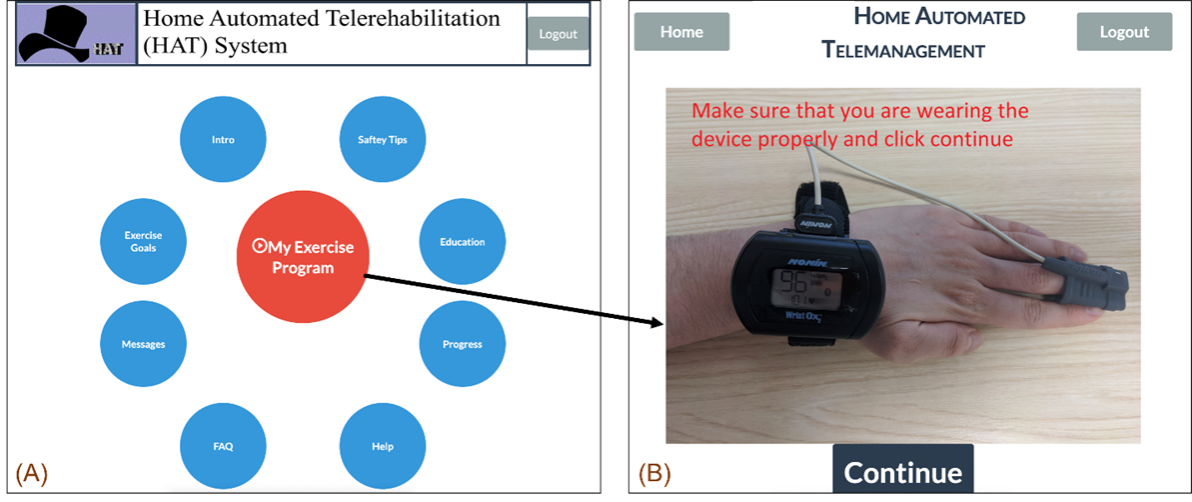
Main menu of the Home Automated Telemanagement (HAT) patient interface. The figure shows the user-friendly interface that allows patients to access their personalized exercise program, symptom monitoring, and communication tools with their clinicians. This interface is designed for patients with limited mobility, vision, or cognitive impairments, offering multiple input methods, including touch screen, mouse, and speech. Patients can start their exercises, track progress, and communicate directly with their health care providers through this interface.

**Figure 3:**
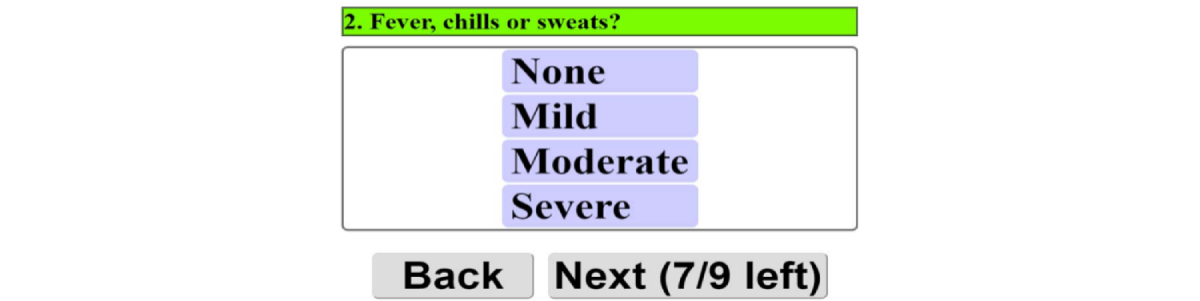
Example of the symptom diary in the Home Automated Telemanagement (HAT) system. The symptom diary allows patients to log their symptoms before starting their prescribed exercise program. The system uses these inputs to determine the appropriateness of exercise based on the patient's health status.

**Figure 4:**
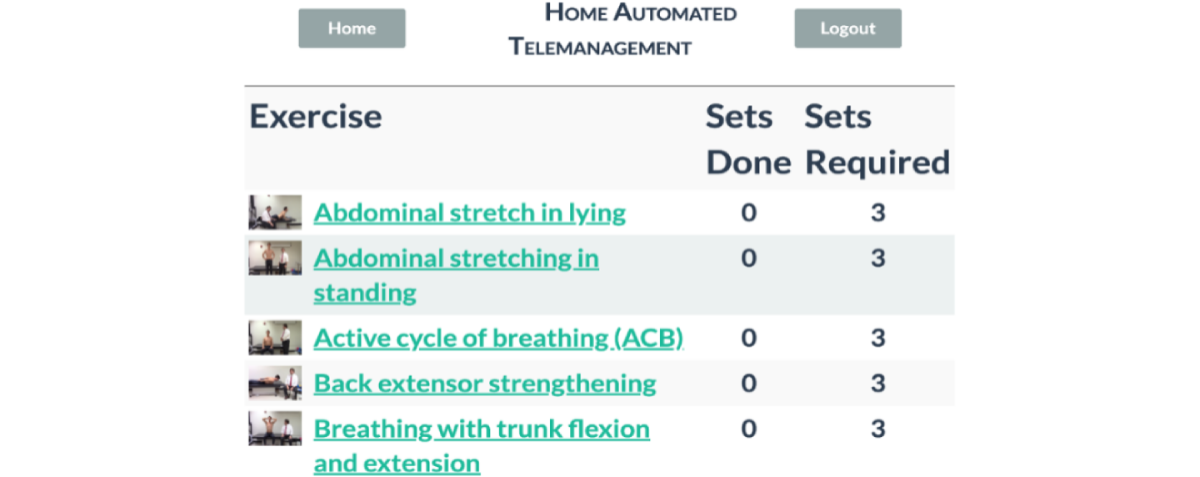
Patient exercise list in the Home Automated Telemanagement (HAT) system. This figure displays the list of prescribed exercises a patient can choose from within the HAT system's patient interface. Each exercise is customized based on the patient's specific health condition and rehabilitation goals, which the clinician prescribes. The system includes step-by-step instructions and precautionary guidelines for each exercise. The list is tailored to each patient, ensuring that exercises match their abilities and health status.

Patients can log on and complete a prescribed individually tailored exercise program using the patient portal. The main menu is shown in Figure 2A. The figure shows the user-friendly interface that allows patients to access their personalized exercise program, monitor symptoms, and communicate with their clinicians. This interface is designed for patients with limited mobility, vision, or cognitive impairments, offering multiple input methods, including touch screen *and* mouse. Patients can start their exercises, track progress, and communicate directly with their health care providers through this interface. Before starting the prescribed activity, the patient is asked to wear a pulse oximeter device to ensure the oxygen saturation level and heart rate are in the normal range (Figure 2B). The patient is then asked a series of questions about their current symptoms. Figure 3 shows an example of the questions. The symptom diary allows patients to log their symptoms before starting their prescribed exercise program. The questions are designed depending on the type of patient’s disease. The remainder of the exercise is prohibited if the patient’s symptoms are too severe. They are given a list of all recommended exercises, as illustrated in Figure 4, if they fall within the symptom severity threshold. The list of prescribed exercises a patient can choose from within the HAT system’s patient interface is shown in Figure 4. Each exercise is customized based on the patient’s specific health condition and rehabilitation goals, which the clinician prescribes. The system includes step-by-step instructions and precautionary guidelines for each exercise. The list is tailored to each patient, ensuring that exercises match their abilities and health status.

**Figure 5:**
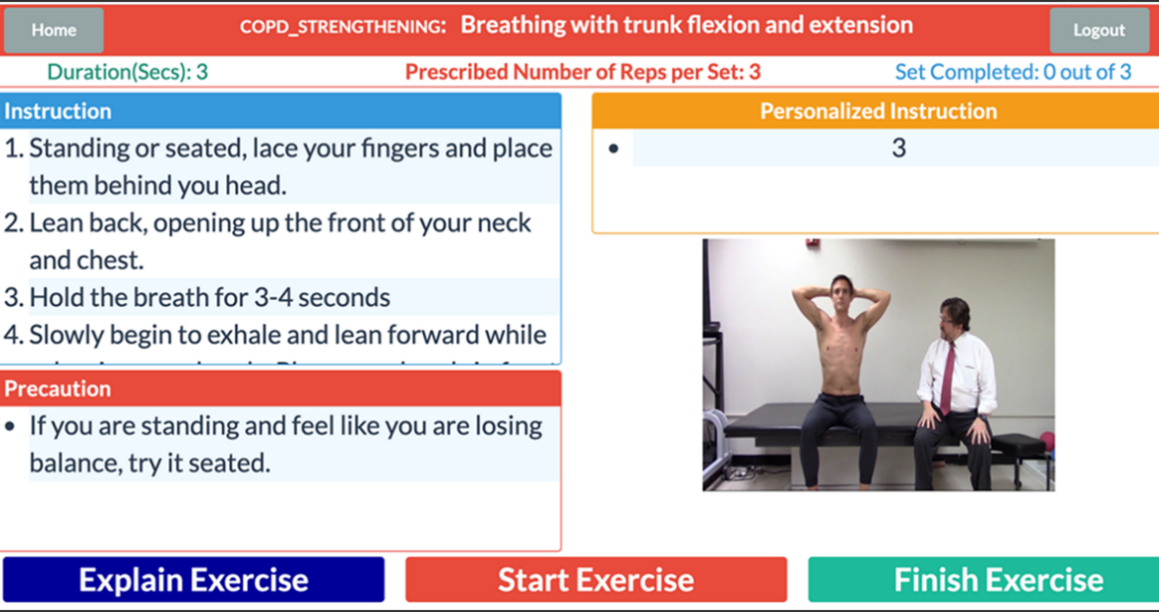
Exercise screen in the Home Automated Telemanagement (HAT) system. This figure shows the real-time exercise monitoring screen that patients interact with during their prescribed exercise sessions. The screen provides patients with live feedback on their performance, including the exercise duration, cycling speed, and progress toward the exercise goal. It also includes visual and numerical indicators to guide patients in maintaining the correct pace according to the guidelines prescribed by their clinician. This interface ensures that patients can safely perform their exercises at home. At the same time, the system collects performance data and sends them to clinicians for remote monitoring and adjustment of future sessions.

After deciding on an exercise, the patient receives step-by-step exercise instructions and precautions. They can also view a video demonstrating how to perform the activity. When the patient is prepared to begin the exercise, they select “Start Exercise.” At this point, a video of the exercise repetitions is played, and the number of repetitions is counted. The patient clicks “Finish Exercise” after completing the required repetitions. Then, the patient is asked specific postexercise questions about the completed exercise. An example of the exercise screen is shown in Figure 5. This figure shows the real-time exercise monitoring screen that patients interact with during their prescribed exercise sessions. The screen provides patients with live feedback on their performance, including the exercise duration, cycling speed, and progress toward the exercise goal. It also includes visual and numerical indicators to guide patients in maintaining the correct pace according to the guidelines prescribed by their clinician. This interface ensures that patients can safely perform their exercises at home. The HAT server receives all survey results and workout progress data in real time, which are then made available for inspection on the clinician site. A video introduction, safety tips, interactive education, exercise progress, exercise goals, messages, frequently asked questions, and a help section are also included in the patient site. The patient can message the clinician, track their progress, and review personalized goals.

### Provider Unit

**Figure 6:**
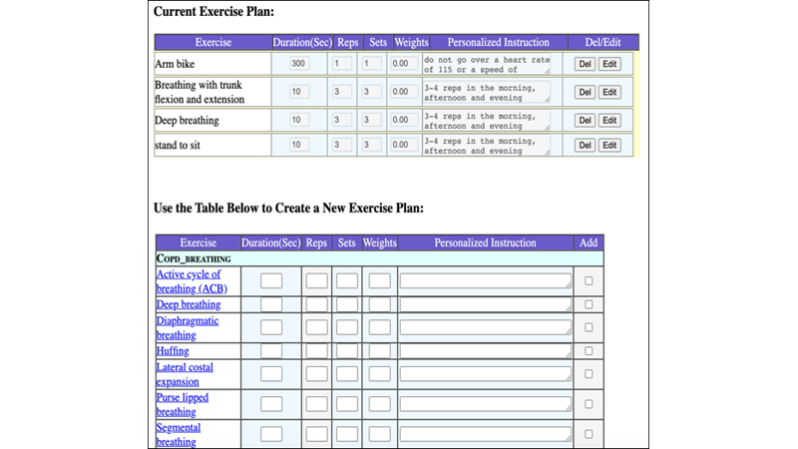
Editing patient exercises in the Home Automated Telemanagement (HAT) provider interface. This figure displays the clinician's interface for modifying a patient's exercise program within the HAT system. Clinicians can set or adjust parameters tailored to each patient's rehabilitation needs, such as exercise time, repetitions, sets, and weights. This feature allows for ongoing customization of the exercise regimen based on the patient's progress and feedback.

**Figure 7:**
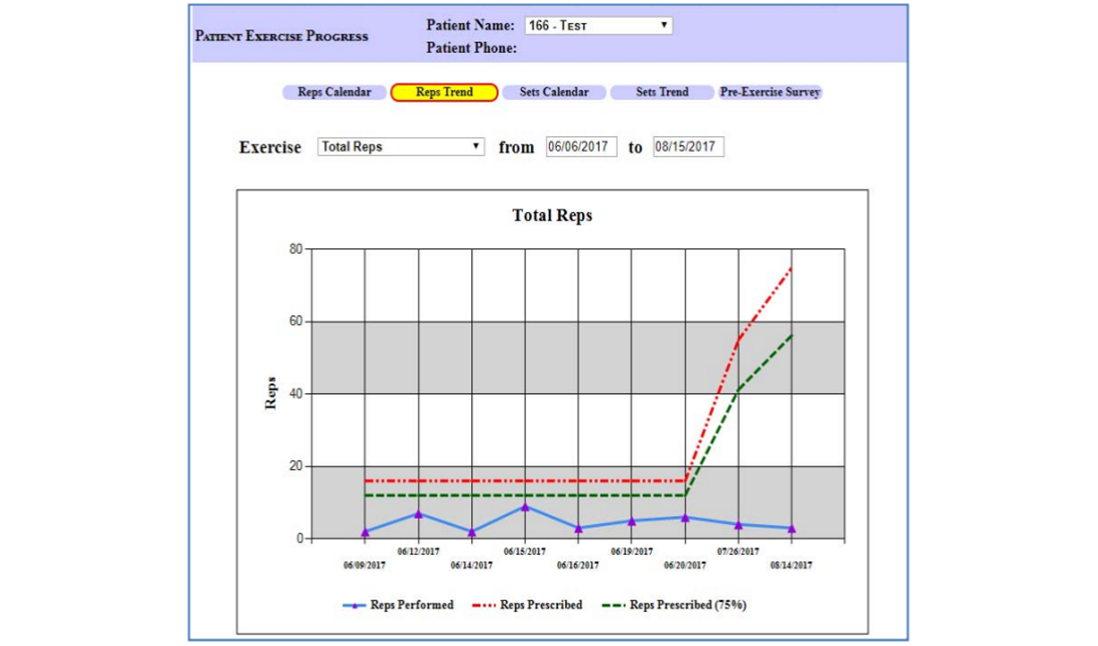
Exercise progress monitoring in the Home Automated Telemanagement (HAT) system. This figure presents the clinician's view of a patient's exercise progress. The system tracks metrics such as exercise frequency, duration, and compliance with prescribed guidelines, displaying trends that help clinicians evaluate the effectiveness of the prescribed regimen. This feedback enables timely adjustments to the patient's exercise program and supports data-driven decision-making.

The HAT provider site enables the rehabilitation team to alter the patient’s exercise plan, view exercise and survey reports, set alert levels, communicate with the patient, prescribe an exercise program, and set exercise targets. Based on the patient’s performance status, the rehabilitation team can update the patient’s exercise program. For each workout, they can specify the time, repetitions, sets, weights, and individualized instructions. This feature allows for ongoing customization of the exercise regimen based on the patient’s progress and feedback. Figure 6 displays the menu for editing exercises. The HAT provider can also see the outcomes of earlier exercises. The patient can access the results immediately after finishing the test. As seen in Figure 7, trends can be observed for specific periods. The system tracks metrics such as exercise frequency, duration, and adherence to prescribed exercises, displaying trends that help clinicians evaluate the effectiveness of the prescribed regimen. This feedback enables timely adjustments to the patient’s exercise program and supports data-driven decision-making.

**Figure 8:**
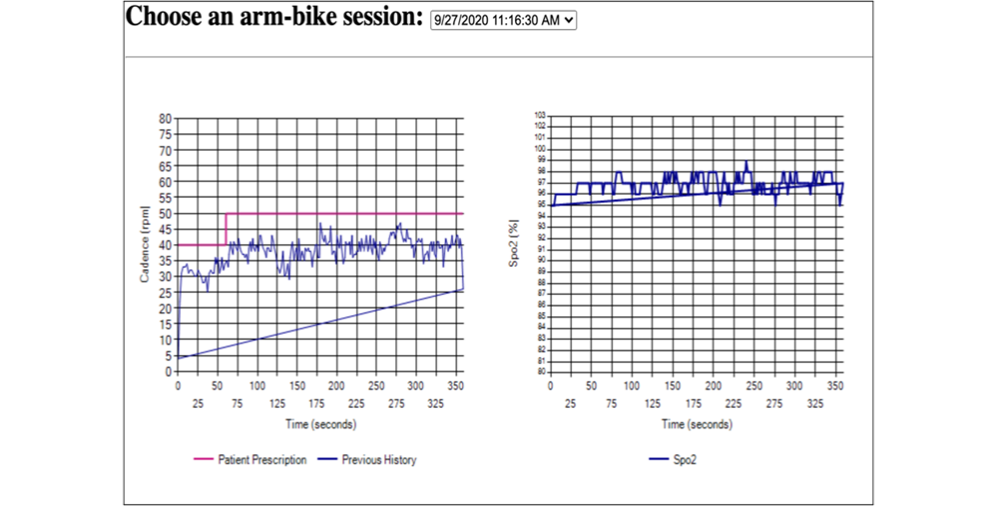
Arm bike progress and oxygen saturation (SpO_2_) level during exercise. This figure illustrates 2 key metrics monitored during the arm bike exercise session: progress toward the exercise goal (left plot) and SpO_2_ levels (right plot). Target speed intervals guide the arm bike exercise, and the system ensures that patients maintain the prescribed pace. Monitoring SpO_2_ levels ensures patient safety.

**Figure 9:**
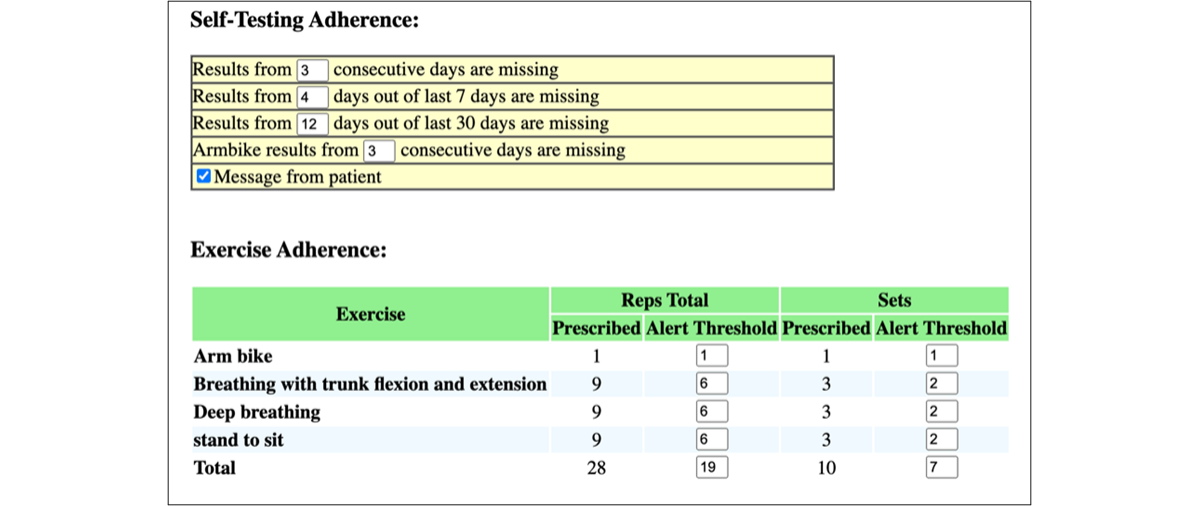
Alert parameters for patient monitoring in the Home Automated Telemanagement (HAT) system. This figure shows the interface where clinicians can set alert parameters for monitoring patient exercise adherence and safety. These alerts are triggered if the patient deviates from the prescribed exercise plan or if specific thresholds (such as heart rate or exercise frequency) are unmet. Clinicians can review these alerts daily and adjust the patient's program as needed.

**Figure 10:**
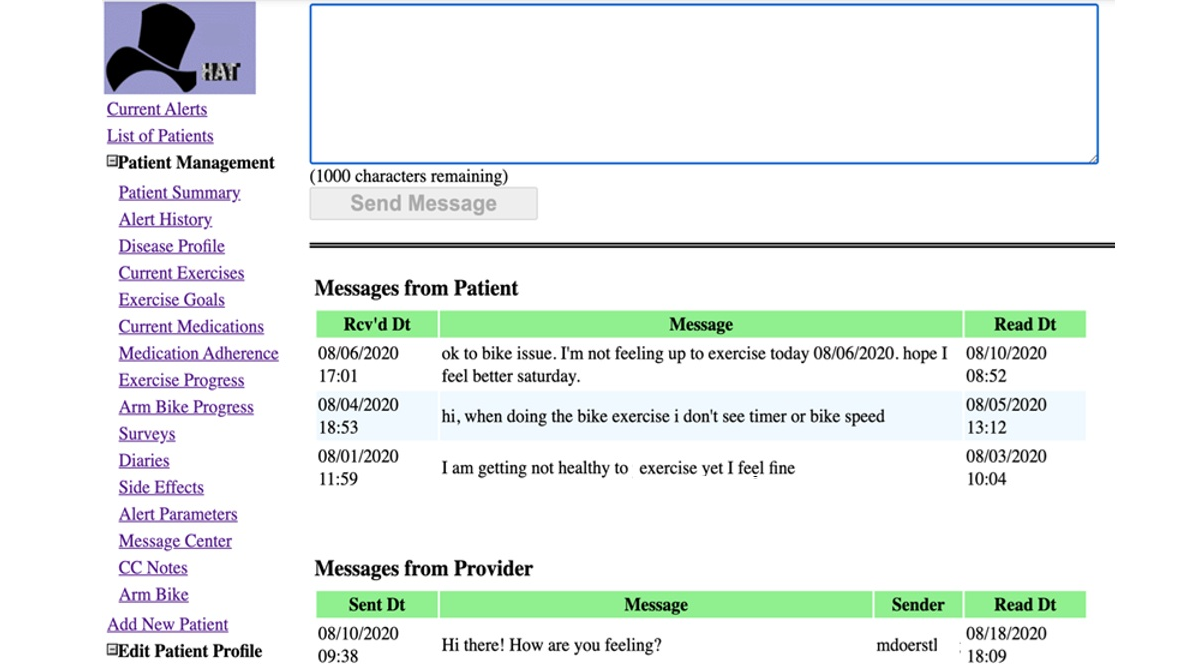
Clinician-Patient Messaging System in the Home Automated Telemanagement (HAT) platform. This figure illustrates the messaging feature that enables direct communication between the clinician and the patient within the HAT system. Clinicians can send personalized exercise recommendations, answer questions, or provide encouragement, while patients can report any issues or ask for clarification. This 2-way communication is integral to maintaining patient engagement and promptly addressing any issues.

In addition to the initially prescribed personalized exercise plan, provided by the physicians and therapists, the providers can review the outcomes of earlier workouts and update the exercise plan as needed according to objective patient data (Figure 8). During the session, the patient strives to adhere to the time and speed intervals that the clinician assigns (the red line in Figure 8). Customized alerts tailored to specific patient profiles can be established by the provider. These notifications are issued every morning and can reflect patient communications, exercise adherence, and patient symptoms. When the provider logs in, generated alerts appear in the list of current alerts. The rehabilitation team identifies clinically actionable alerts and responds to them by adjusting the exercise plan, contacting the patients or primary and specialty providers caring for the patients. Each alert is deactivated only after the provider documents the response in the alert dashboard. In Figure 9, the alert parameters are displayed. On the provider’s portal, the provider can also set exercise objectives for their patients, communicate with them, see their survey results, and take notes. In addition, the clinician and patients can exchange messages (Figure 10). This 2-way communication is integral to maintaining patient engagement and promptly addressing any issues.

### iBikE Unit

**Figure 11:**
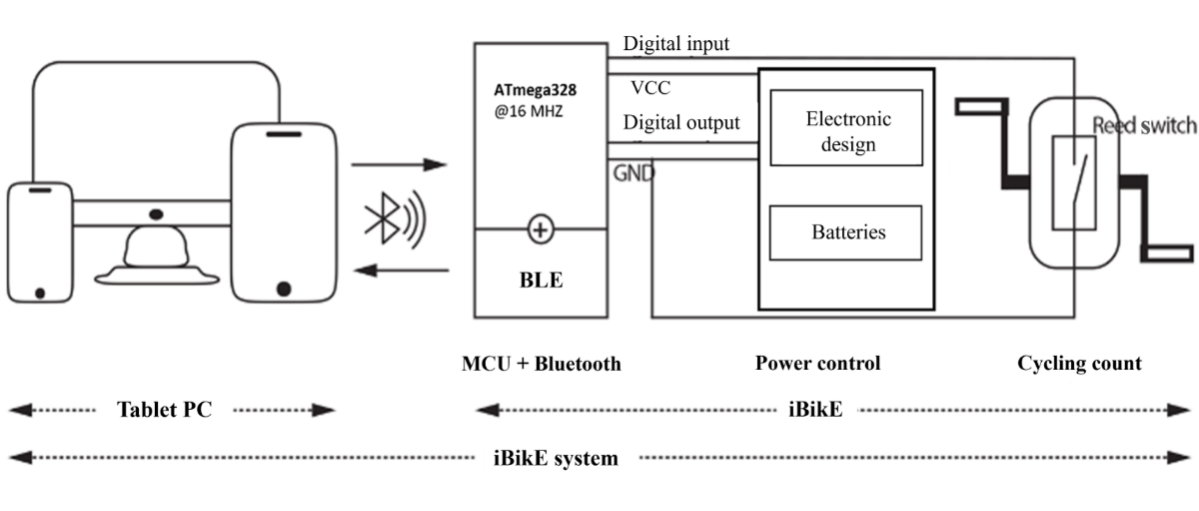
Interactive bike (iBikE) system design. This figure outlines the technological components of the iBikE system, which is integrated into the Home Automated Telemanagement (HAT) platform. The system comprises a Bluetooth-enabled cycling device (iBikE) and a tablet for real-time data display and user interaction. The figure shows how the iBikE captures cycling data via a magnetic switch and transmits this information to the HAT system. BLE: Bluetooth Low Energy; MCU: microcontroller unit; VCC: voltage common collector.

The iBikE functionality was successfully integrated with the HAT system to support the arm bike exercise program. The technical aspects of the bike are explained in [26]. The iBikE system utilizes (1) a tablet, user interface, and data logging; and (2) iBikE exercise equipment, as shown in Figure 11. This figure shows the technological components of the iBikE system, which is integrated into the HAT platform.

**Figure 12:**
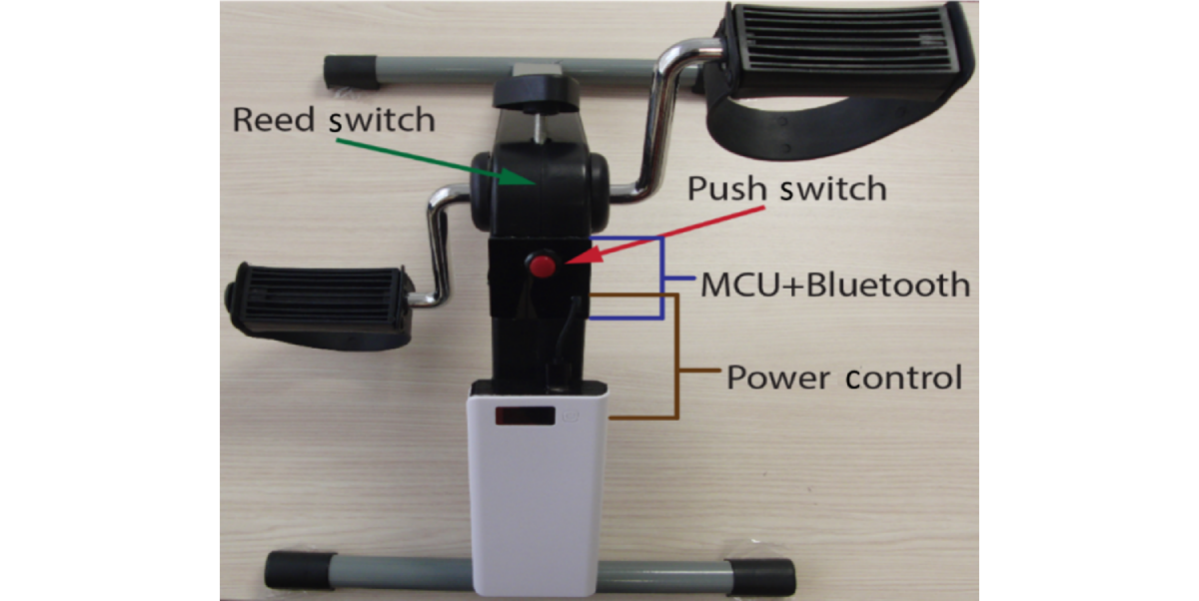
Interactive bike (iBikE) exercise equipment. This figure shows the Home Automated Telemanagement (HAT) system's iBikE exercise equipment used for arm and leg cycling exercises. The equipment is lightweight, portable, and connected to the system via Bluetooth, allowing real-time tracking of the patient's cycling speed and compliance with the prescribed exercise guidelines. The iBikE's compact design makes it suitable for home use, supporting rehabilitation for patients with mobility issues. MCU: microcontroller unit.

The touch screen–operated operator interface was developed using the LabVIEW graphical programming environment (National Instruments Corporation). The program used Bluetooth communication technology to send and receive data and display real-time information on the screen. The iBikE exercise equipment is shown in Figure 12. The equipment is lightweight and portable. Its compact design makes it suitable for home use, supporting rehabilitation for patients with mobility issues. The iBikE in this study was created using Bluno Beetle (DFRobot, an Arduino Uno–Based board with Bluetooth 4.0 [Bluetooth Low Energy]). The iBikE first reads the data from the magnetic switch module via the microcontroller unit to retrieve cycle information. Accurate real-time cycling data were calculated using the algorithm defined for the microcontroller unit. The tablet received real-time cycling data from the iBikE via Bluetooth.

The magnetic switch allows to record the amount of time needed to cycle iBikE once. The magnet switch is an apparatus that transforms variations in magnetic fields into electrical impulses. The device’s magnetic switch recognizes a change in the magnetic field when the magnet gets close to it. The magnetic switch picks up another change when the magnet leaves the magnetic field.

These changes formed an electrical circuit to detect on/off signals. The sensor’s contact was identified once during each time cycle. Continuous detection of rising and falling edge changes prevented errors in on/off detection. It defined the user’s departure from iBikE if there was no detection of on/off changes for more than 30 seconds. In addition, this cycling information was stored on the HAT server to allow users and medical professionals to share and review it online. The default values of the two 2-pointing windows were set to graphically display the data and displayed on the screen to induce an immediate response to the user’s instructional speed. The speed of the instructions was used for the usability test downloaded on the local server. The set speed of guidance was 75 rpm for the first 3 minutes, 90 rpm for the 1-minute mid-term, and 60 rpm for the last minute.

**Figure 13:**
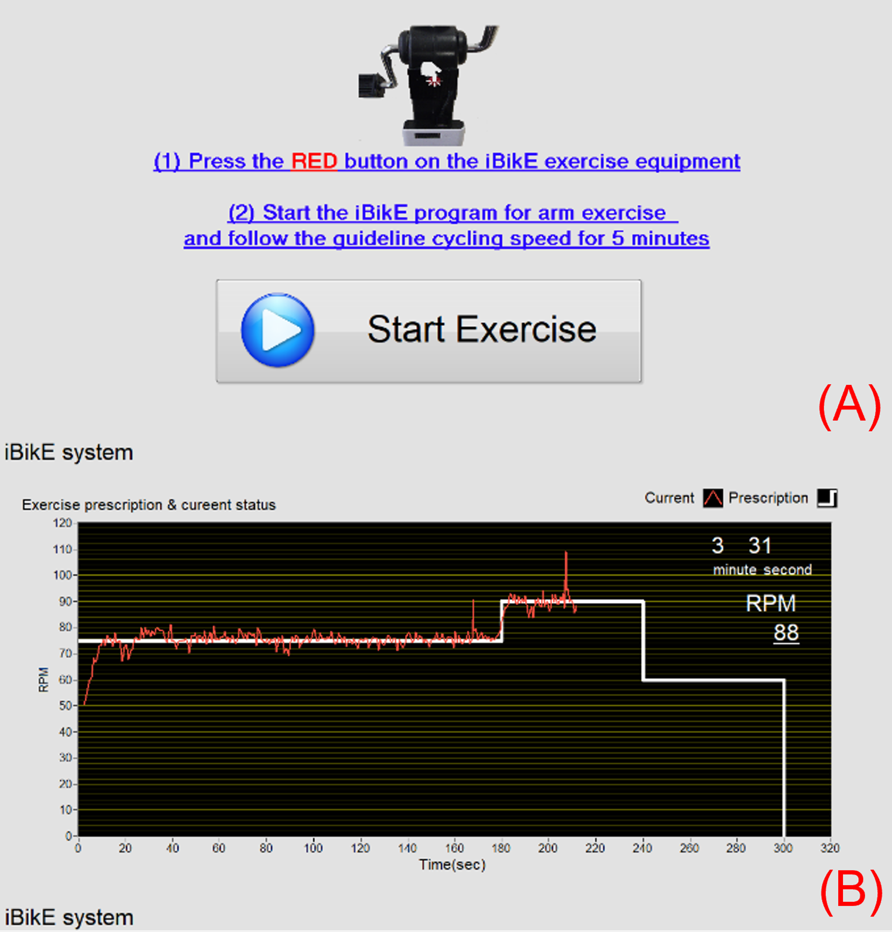
interactive bike (iBikE) program user interface.

The operating procedure required the user to first press the red button on the iBikE exercise equipment to wake it up and apply Bluetooth communication pairing with the tablet. Subsequently, on the first page of the user interface, the user pressed or clicked the button (Figure 13A) to indicate the beginning of the exercise to allow the tablet to connect to the Bluetooth communication pairing with the iBikE exercise equipment. When communications between the tablet and iBikE were paired, the prescribed speed trajectory appeared on the second page of the interface (Figure 13B), and the user could exercise via iBikE exercise equipment and receive real-time feedback on the exercise. In addition, the user’s exercise elapsed time and current cycling speed were shown in numerical format on the screen.

### System Evaluation

#### Demographic Characteristics of the Study Participants

Table 2 summarizes the demographic characteristics of the study participants who tested the iBikE system as part of the HAT system usability evaluation. Participants included adults with an average age of 37.4 (SD 14.2) years. All of the participants reported permanent employment. The table details participants’ internet use, technology habits, and self-reported English proficiency. Multimedia Appendix 1 compares the results of the 2 consecutive usability tests, highlighting the reduction in task completion times and increased satisfaction scores across various metrics. Participants rated their experience on a scale of 1-5, with the majority reporting high satisfaction and ease of use. Participants completed the tasks faster and more accurately during the posttest after a 1-week hiatus, demonstrating the system’s effectiveness in promoting user retention and adaptability in home-based rehabilitation. Table 3 reports the SUS scores for the iBikE system’s pre- and posttests, providing an overall measure of user satisfaction. The scale ranges from 0 to 100. Table 4 includes participant responses to questions about system complexity, confidence in using the system, and the likelihood of recommending the iBikE system to others.

**Table 3:** System Usability Scale scores for the iBikEa system usability testing. This table reports the System Usability Scale scores for the iBikE system’s pre- and posttests, providing an overall measure of user satisfaction. The scale ranges from 0 to 100. The table also includes participants’ responses to questions about system complexity, confidence in using the system, and the likelihood of recommending the iBikE system to others.

Questionnaire			Test 1, mean (SD)		Test 2, mean (SD)
**System Usability Scale (1-5)^b^**					
	I think that I would like to use this system frequently	5.0 (0.0)		5.0 (0.0)	
	I found the system unnecessarily complex	1.0 (0.0)		1.2 (0.5)	
	I thought the system was easy to use	4.8 (0.5)		5.0 (0.0)	
	I think that I would need the support of a technical person to be able to use this system	3.0 (1.2)		1.8 (1.3)	
	I found the various functions in this system were well integrated	5.0 (0.0)		5.0 (0.0)	
	I thought there was too much inconsistency in this system	1.2 (0.5)		1.0 (0.0)	
	I would imagine that most people would learn to use this system very quickly	5.0 (0.0)		5.0 (0.0)	
	I found the system very cumbersome to use	1.0 (0.0)		1.2 (0.5)	
	I felt very confident using the system	5.0 (0.0)		5.0 (0.0)	
	I needed to learn a lot of things before I could get going with this system	1.8 (1.8)		1.0 (0.0)	
**System Usability Scale score (0-100)**			92 (8.6)		97 (3.3)

^a^iBikE: interactive bike.

^b^1=strongly disagree, 2=somewhat disagree, 3=neutral, 4=somewhat agree, and 5=strongly agree.

**Table 4:** Results of the iBikEa attitudinal survey. This table includes participant responses to questions about system complexity, confidence in using the system, and the likelihood of recommending the iBikE system to others.

Questionnaire		Test 1^b^, mean (SD)	Test 2^c^, mean (SD)
**How likely are you to recommend this iBikE system to others? (0-10)^d^**		9.4 (0.9)	9.8 (0.5)
**Attitudinal survey (1-4)**			
	Did you get all the necessary information about using the iBikE system during the initial practice session?^e^	1.0 (0.0)	1.0 (0.0)
	How complicated was it to use the computer?^f^	4.0 (0.0)	4.0 (0.0)
	How difficult was it to use the touch screen/mouse?^g^	4.0 (0.0)	4.0 (0.0)
	Did you have any difficulties in reading the graphic indicator from the dashboard of the iBikE system?^h^	1.0 (0.0)	1.6 (1.3)
	Did you have any difficulties in reading the numerical indicator from the dashboard of the iBikE system?^h^	1.0 (0.0)	1.6 (1.3)
	How complicated was it to complete the provided overall exercise procedure?^i^	1.0 (0.0)	1.0 (0.0)
	How difficult was arm exercising?^j^	1.0 (0.0)	1.0 (0.0)
	Did you get all the necessary information about your exercise status during your exercise?^e^	1.0 (0.0)	1.0 (0.0)
	Would you feel safer while your exercise status (duration and speed) is being monitored during your exercise?^k^	1.6 (0.6)	1.2 (0.5)
	How important for you is it to know that the results can be reviewed by a clinician immediately after your exercise?^l^	1.6 (1.3)	1.8 (1.3)
	Would you like to use this iBikE system in the future?^m^	1.0 (0.0)	1.0 (0.0)
	Would you advise other people to use this iBikE system?^m^	1.0 (0.0)	1.2 (0.5)
	Overall, how would you grade the whole procedure of the study?^n^	1.2 (0.5)	1.0 (0.0)
	Overall, how would you grade the iBikE system?^n^	1.0 (0.0)	1.0 (0.0)
**Exit survey (1-5)^o^**			
	Is the iBikE system an appealing means for home exercise?	5.0 (0.0)	5.0 (0.0)
	Is the iBikE system easy to use?	5.0 (0.0)	5.0 (0.0)

^a^iBikE: interactive bike.

^b^Test 1: n=5.

^c^Test 2: n=5.

^d^0=not at all likely to 10=extremely likely.

^e^1=all information, 2=almost all information, 3=partial information, and 4=very limited information.

^f^1=very complicated, 2=moderately complicated, 3=slightly complicated, and 4=not complicated at all.

^g^1=very difficult, 2=moderately difficult, 3=slightly difficult, and 4=not difficult at all.

^h^1=not at all, 2=a little, 3=considerably, and 4=significantly.

^i^1=not complicated at all, 2=slightly complicated, 3=moderately complicated, and 4=very complicated.

^j^1=not difficult at all, 2=slightly difficult, 3=moderately difficult, and 4=very difficult.

^k^1=significantly safer, 2=moderately safer, 3=maybe safer, and 4=feel nothing.

^l^1=extremely important, 2=very important, 3=important, and 4=maybe.

^m^1=certainly yes, 2=maybe, 3=unlikely, and 4=no.

^n^1=excellent, 2=good, 3=satisfactory, and 4=needs serious improvement.

^o^1=strongly disagree, 2=somewhat disagree, 3=neutral, 4=somewhat agree, and 5=strongly agree.

#### Usability Test 1 (Pretest)

The introduction phase of the iBikE system required a mean of 196.2 seconds. The users could complete all tasks without any help during the tasks. High positive mean scores of 5 were given in the 3-item posttask survey for task 1.1 and task 2.2, and 4 and higher were given for task 1.2 (mean 4.8, SD 0.5), task 1.3 (mean 4.6, SD 0.6), task 2.1 (mean 4.4, SD 1.3), and task 2.3 (mean 4.4, SD 1.3). Detailed session information, times to accomplish tasks, and posttask questionnaire scores are shown in Multimedia Appendix 1 and Table 3. An attitudinal survey reflected a high overall acceptance of guided exercise biking (Table 4). Based on the SUS results, patients felt the system was easy to use. For the SUS, the mean of the usability scale was 92 (SD 8.6; range 0-100).

#### Usability Test 2 (Posttest)

The introduction phase of the iBikE system was not performed because the posttest wanted to assess the pure usability of this system after the first week of use. No help during any of the sessions was needed. The users were able to complete all tasks. High positive mean scores of 5 were given in the 3-item posttask survey for task 1.1, task 2.2, and task 2.3, and 4 and higher were given for task 1.2 (mean 4.8, SD 0.5), task 1.3 (mean 4.8, SD 0.5), and task 2.1 (mean 4.8, SD 0.5). Detailed session information, times to accomplish tasks, and posttask questionnaire scores are shown in Multimedia Appendix 1 and Table 3. An attitudinal survey reflected a high overall acceptance of guided exercise biking (Table 4). The SUS results showed that the patients felt the system was easy to use. For the SUS, the mean of the usability scale was 97.0 (SD 3.3; range 0-100).

#### Comparison of Pre-Post Tests

The completion time for each task, measured during sequential usability tests, was recorded without prior training after 1 week. The completion times of task 1 were changed from a mean of 8.6 (SD 4.7) seconds to a mean of 1.8 (SD 0.8) seconds, and the accomplished times of task 2 were changed from a mean of 315.0 (SD 6.9) seconds to a mean of 303.4 (SD 1.1) seconds (Multimedia Appendix 1). The 3-item posttask survey scores increased from task 1.3, task 2.1, and task 2.3. The attitudinal survey between pre-post tests showed an increase in scores in questions of “Did you have any difficulties in reading the graphic indicator from the dashboard of the iBikE system?” “Did you have any difficulties in reading the numerical indicator from the dashboard of the iBikE system?” “How important for you is it to know that the results can be reviewed by a clinician immediately after your exercise?” and “Would you advise other people to use this iBikE system?” and a drop in scores in questions of “Would you feel safer while your exercise status (duration and speed) is being monitored during your exercise?” and “Overall, how would you grade the whole procedure of the study?” The scores for the SUS were increased from a mean of 92 (SD 8.6) to a mean of 97 (SD 3.3; Table 3). From the question “How likely are you to recommend this iBikE system to others?” the scores increased from a mean of 9.4 (SD 0.9) to a mean of 9.8 (SD 0.5; Table 4). Table 5 presents the differences between the performed cycling speed and the guideline prescribed speed for 5 participants during 2 usability tests (pre- and posttest). As shown in Table 5, the speed compliance (SD of [current speed–guideline speed]) provided during the 5-minute arm exercise was found to be reduced from the pretest (mean 6.26 rpm, SD 1.00 rpm) to the posttest (mean 4.02 rpm, SD 0.82 rpm; *t=*3.305, n=5, *P*=.03). Problems found by users through 2 tests and suggestions and comments after using the system are shown in Textbox 3.

**Table 5:** Differences between performed speed and guideline speed for iBikEa exercise sessions. This table presents the differences between the performed cycling speed and the prescribed speed for 5 participants during 2 usability tests (pre- and posttest). These findings indicate improved compliance with the guideline speed after using the iBikE system for 1 week.

User	Mean of errors^b,c^ (rpm^d^)		SD of errors^c,e^ (rpm^d^)	
	Test 1^f^	Test 2^g^	Test 1^f^	Test 2^g^
1	–0.3	–0.9	5.9	3.0
2	–3.3	0.4	8.0	3.7
3	0.7	0.6	6.1	3.9
4	0.1	1.0	5.7	4.3
5	–1.3	–1.0	5.6	5.3
Mean (SD)	–0.83 (1.57)	0.03 (0.88)	6.26 (1.00)^h^	4.02 (0.82)^h^

^a^iBikE: interactive bike.

^b^Mean of errors=(Σ[*t*=0seconds~300seconds] error of each revolution)/number of total revolutions.

^c^Error: performed speed-guild-line speed.

^d^rpm: revolutions per minute.

^e^SD of errors=SD of (Σ[*t*=0seconds~300seconds] error of each revolution).

^f^Test 1: n=5.

^g^Test 2: n=5.

^h^Paired *t*=3.305, n=5, *P*=.03.

**Usability issues, comments, and suggestions for the interactive bike (iBikE) system. This table summarizes the feedback provided by participants after 2 usability tests of the iBikE system. These insights provide valuable feedback for refining the system design to improve user experience and exercise adherence.**

**Problems users encountered while using the interactive bike (iBikE) system.**

1. Test 1
  - As the equipment is lightweight, it is necessary to consider a mounting fixture or method that secures the portable bike during user exercise.
  - The equipment, if not adequately secured, might move and fall off from the desk during arm cycling exercise.
  - It would be great if patients could only click the red button once and start the exercise without additional steps.
  - The equipment needs to be fixed in a secure position on the table during exercise using a rubber mat.
2. Test 2
  - It may be hard to stay within the prescribed cycling speed. The displayed speed is too sensitive to minor changes in cadence.
  - The graphical dashboard and lines presenting the prescribed and current cycling speed should be larger so it is easier to follow the speed prescription during actual biking exercises.
  - The interface should include help functionality explaining the bike operations and examples with corresponding video clips.

## Discussion

### Principal Findings

The presented HAT system supports individualized multipronged in-home exercise programs for patients requiring physical rehabilitation services. We prepared and tested a comprehensive system that provides patient and physician feedback. Our previous studies have shown that patients with ulcerative colitis using the HAT system had improved disease control, fewer hospitalizations, and increased quality of life [29]. The system was found to be useful in monitoring symptoms and disease progression for patients with multiple sclerosis [30,31]. The HAT system was also well accepted and considered easy to use by the study participants [31-33]. We also added the iBikE system to the HAT system to show its capability to adopt resources that can be quickly accessible for targeted patients. Patients can use the system and follow their personalized exercise prescription. The system also assists patients at home in following their program safely and effectively. In addition, they can easily communicate with their physicians. Patients’ data could be recorded in the HAT database for further evaluation. Physicians can monitor patients’ progress over time and decide to change or modify their prescriptions.

We also evaluated the effects of the new iBikE system on users by assessing their satisfaction. The study showed positive, relevant changes in early users using the system for telerehabilitation. Users were very satisfied with the system and encouraged to use actual patients. We found average user satisfaction and adaptability improvements from the pre-post tests conducted without additional user exposure in 1 week (Multimedia Appendix 1 and Tables 3 and 4). When evaluating user exercise adherence for the given prescribed speed, we observed that the users substantially improved adherence to the prescribed speed at the second visit by approximately 2 rpm over the initial visit (Textbox 3). This change indicated that users could show sustainable adaptability in following the exercise protocol and maintaining the skills to operate the system successfully over time. No interruptions of exercise performance due to errors in the system use were observed during the second visit. The user satisfaction with the system functionality and interface was very high (Table 3). The users provided valuable input to be addressed in the next iterations of the system.

Our results showed a high level of system acceptance for participating users. The average introductory process of about 3 minutes was enough time for users to understand how to use the system and prepare for it independently (Multimedia Appendix 1). After the usability test’s introduction, all users could complete the tasks necessary for their exercise programs. The posttest, which took place a week later, also reduced the time required to perform the pretest despite omitting this introduction process. Previous studies have shown considerable potential for telerehabilitation approaches that support home-based exercises. However, they mainly focused on balancing and walking corrections and did not include consideration for users with mobility disabilities [5]. One of the system’s main advantages is that it can help users overcome time and location problems by providing a platform that allows them to continue to access exercises for rehabilitation at home without having to visit hospitals or rehabilitation facilities. This can lead to longer-term exercise adherence, including for users with mobility limitations.

Two repeated system usability evaluations measured from the same user have yielded promising preliminary results on telerehabilitation exercise devices using this system. The pre and posttests evaluated whether the same or different problems occurred among users. This method could be a valuable tool for enhancing the science of usability testing. Although we only adopted a series of test cycles that required testing to be repeated until further usability problems were identified, we identified system improvements using repeated feedback from all users in the one-off cycle. The survey enabled us to work with the users to identify the necessary elements to ensure that the system was suitable. It also provided insights into potential barriers to implementation. Users’ responses provided information about the functions expected by the iBikE system, which would directly impact the design of the next version of the iBikE system. In addition to conducting usability testing, user participation continued through direct in-person interviews. Usability testing will result in updates to the system implementation that can be deployed in real-world environments.

Generally, users wanted a simple easy-to-use system. They were satisfied with the purpose and content of the developed prototype and agreed that it was a helpful resource for people who needed home rehabilitation. Users in the usability test session found the developed system to be a valuable and easy-to-use exercise program that supported the user’s confidence in performing home-based exercise according to the individually prescribed exercise plans. User feedback highlighted the need to secure exercise equipment to the floor or table, have a more straightforward way to connect to Bluetooth communication between the exercise equipment and the tablet, and provide better visualization of the exercise’s progress and remaining exercises. They also mentioned an increase in the font size of numeric and graphical indicators.

One of the primary limitations of this study is the relatively small sample size. The limited number of participants may reduce the statistical power of the findings, potentially leading to results that are less robust or representative of a broader population. Although the number of participants was limited, it was sufficient to identify usability problems [34]. Future work should include a larger sample of diverse individuals testing the system for a prolonged period. Both long- and short-term effects should be investigated and compared with the existing rehabilitation effects implemented in outpatient clinics. We plan to conduct additional tests in proof-of-concept studies to address the users’ feedback and ensure the high fidelity, reliability, and usability of the system.

### Conclusions

In summary, this study demonstrates the potential efficacy of the iBikE system in enhancing patient engagement and promoting individualized exercise programs through telerehabilitation. The key findings indicate that the system significantly improved both the usability and adherence of participants, contributing to positive patient outcomes, including increased physical activity and motivation. These results suggest that the iBikE system has the potential for broader application in home-based rehabilitation programs, particularly for individuals with mobility limitations or those requiring long-term exercise interventions.

It is important to note that future research should focus on validating the findings of this study by expanding it to larger and more diverse populations. Further assessment of the system’s impact on long-term health outcomes is necessary using a randomized controlled trial design. Additionally, exploring the integration of more advanced features, such as real-time feedback and personalized data tracking, could enhance its effectiveness in telerehabilitation. Overall, the iBikE system represents a promising tool in the field of home-based rehabilitation, offering a scalable solution for improving patient outcomes in remote settings.

Further development of the HAT system should include support for treatment adherence [35], virtual tele-visits by therapists and caregivers [36], interactive health education and empowerment [37], an extension of exercise options [38,39], automated assessment of levels of exercise exertion [40], social exergaming [41], the inclusion of cardiovascular monitoring [42], and cuff-less measurement of blood pressure during exercise [43]. Future telerehabilitation systems will utilize the Internet of Things (IoT) architecture to achieve interoperability [44] and seamless inclusion of a broad spectrum of wearable, ambient, and contactless sensors [45,46], which will generate rich data sets analyzed by artificial intelligence to optimize exercise programs [47] and engage patients in interactive experience using virtual reality [48]. The patient’s needs and preferences should be fully reflected in the next iteration of the telerehabilitation system to achieve high acceptance in the home environment. As a result, the system will have to undergo a comprehensive systematic evaluation in randomized clinical trials to prove its clinical impact.

## Appendix

Multimedia Appendix 1 Comparison of task completion times and user satisfaction: pre- and posttest results for the iBikE system. This table compares the results of the 2 usability tests, highlighting the reduction in task completion times and increased satisfaction scores across various metrics. Participants completed the tasks faster and more accurately during the posttest after a 1-week hiatus, demonstrating the system’s effectiveness in promoting user retention and adaptability in home-based rehabilitation. iBikE: interactive bike.

## Abbreviations

HAT: Home Automated Telemanagement
iBikE: interactive bike
rpm: revolutions per minute
SUS: System Usability Scale
