# Supplementary material for: Home Automated Telemanagement System for Individualized Exercise Programs: Design and Usability Evaluation
Source: JMIR Biomed Eng. 2024 Dec 27;9:e65734. doi: 10.2196/65734 (PMC11724215; doi:10.2196/65734)
Supplement: Multimedia Appendix 1 [file biomedeng_v9i1e65734_app1.docx]

| Session | Accomplished time (seconds), mean (SD) | | Subsession | Scale, mean (SD) | |
| --- | --- | --- | --- | --- | --- |
|  | Test 1^b^ | Test 2^c^ |  | Test 1 | Test 2 |
|  |  |  |  |  |  |
| Introduction | 196.2 (11.7) | N/A^d^ | N/A | N/A | N/A |
| Task 1 | 8.6 (4.7) | 1.8 (0.8) | Task 1.1 | 5.0 (0.0) | 5.0 (0.0) |
|  |  |  | Task 1.2 | 4.8 (0.5) | 4.8 (0.5) |
|  |  |  | Task 1.3 | 4.6 (0.6) | 4.8 (0.5) |
| Task 2 | 315.0 (6.9) | 303.4 (1.1) | Task 2.1 | 4.4 (1.3) | 4.8 (0.5) |
|  |  |  | Task 2.2 | 5.0 (0.0) | 5.0 (0.0) |
|  |  |  | Task 2.3 | 4.4 (1.3) | 5.0 (0.0) |

^a^Both tasks (test 1 and test 2) were accomplished 100% with 0% help needed.

^b^Test 1: n=5.

^c^Test 2: n=5.

^d^N/A: not applicable.
